# Sex of surgeons and team composition and patients’ length of stay after surgery: evidence from inpatient claims data in China

**DOI:** 10.1186/s12916-026-05053-x

**Published:** 2026-07-08

**Authors:** Chi Shen, Yan Zhuang, Shamma Adeeb Alam, Xianhua Zai

**Affiliations:** 1 School of Medicine and Health Management, Tongji Medical College, Huazhong University of Science and Technology, Wuhan, 430030 China; 2 Research Centre for Rural Health Service, Key Research Institute of Humanities & Social Sciences of Hubei Provincial Department of Education, Wuhan, 430030 China; 3 School of Public Health, Health Science Center, Xi’an Jiaotong University, Xi’an, 710061 China; 4 Department of Economics, Dickinson College, 28 N College St, Carlisle, PA 17013 USA; 5 Department of Labor Demography, Max Planck Institute for Demographic Research, 1 Konrad-Zuse-Str, 18057 Rostock, Germany

## Abstract

**Background:**

High-income country evidence suggests female physicians may achieve equal or better patient outcomes, but little is known about how surgeon sex and care-team gender composition affect postoperative recovery in low- and middle-income countries (LMICs). Hospital length of stay after surgery (LOSAS) is a key indicator of recovery efficiency and resource use. We assessed whether surgeon sex and team sex composition are associated with LOSAS in a large LMIC dataset.

**Methods:**

We analyzed administrative data on 602,608 surgical inpatient admissions from all general hospitals in a western Chinese province (January–December 2023). Records were linked with workforce data to identify surgeon sex and team characteristics. Poisson models estimated associations between surgeon sex, team sex composition, and LOSAS, adjusting for patient- and surgeon-level factors, surgery-type and hospital-month fixed effects, enabling comparisons for identical procedures in the same hospital and month. Post-hoc analyses estimated predicted LOSAS in days.

**Results:**

Within the sample, 48.4% (*N* = 363,659) of patients were female. Among the total surgeon population (*N* = 11,994), 58.7% (*N* = 7,042) were female. Patients treated by female surgeons had significantly shorter LOSAS relative to those treated by male surgeons in the overall sample (coefficient = -0.017; SE 0.008). These results remained robust across complex scenarios, including emergency department admissions and cases involving multiple surgeries within a single admission. Furthermore, procedures performed by female surgeons assisted by female first assistants had significantly shorter LOSAS (4.73 days) compared to male–male and female–male teams (5.04 days for both pairings). Younger female surgeons (born 1996–2005) had shorter LOSAS (4.50 days), relative to male counterparts (5.13 days).

**Conclusions:**

In this LMIC setting, female surgeons are associated with shorter LOSAS, even when accounting for case complexity. However, team sex composition and generational differences are influential factors. Our findings suggest shorter LOSAS in female-led teams, specifically those with a female surgeon and a female first assistant, as well as among younger female cohorts. Supporting integration of female surgeons and providing opportunities for female-led teams may therefore enhance recovery efficiency. These results highlight the clinical value of increasing female representation in the surgical workforce.

**1) What is already known on this topic:**

Studies from high-income countries have found that female physicians frequently achieve outcomes equal to or better than their male counterparts in measures such as mortality, readmission, and patient satisfaction. However, the bulk of this work focuses on general medicine rather than operative surgical care, and there is only very limited evidence on how surgeon sex, or the gender composition of surgical teams, influences recovery outcomes in low- and middle-income country (LMIC) settings. Existing studies have also relied primarily on hospital-level analyses, with limited ability to isolate differences by types of surgery or seasonality in effects within hospitals.

**2) What this study adds:**

Leveraging administrative data on over 600,000 surgical cases from a province in China, this study finds that female surgeons are associated with shorter hospital stays, even when accounting for case complexity. Crucially, the research identifies that female-led teams, consisting of a female surgeon and a female first assistant, achieve shorter stays than male-male pairings. Furthermore, the findings reveal a generational shift, where female surgeons in the youngest birth cohort (1996–2005) exhibit shorter recovery times relative to their male counterparts. Methodologically, the study strengthens this literature by utilizing the largest dataset to date from an LMIC setting, providing the statistical power necessary to conduct nuanced subgroup analyses and draw robust inferences. Moreover, our study addresses potential confounding factors by incorporating surgery-type and hospital-month fixed effects. This provides a greater granular level of control than is typically found in the literature; when combined with detailed indicators for surgical complexity and emergency status, it offers robust evidence that gender composition and generational context are critical determinants of surgical outcomes.

**3) How this study might affect research, practice or policy:**

The results suggest that achieving surgical efficiency in resource-constrained health systems may depend on more than simply increasing female surgeon representation. Health systems should consider how team gender composition and generational dynamics shape intraoperative communication, coordination, and patient recovery. For policy makers, this implies that workforce planning should incorporate gender diversity in surgical team design.

**Supplementary Information:**

The online version contains supplementary material available at 10.1186/s12916-026-05053-x.

## Background

Over the past few decades, the physician workforce has undergone substantial changes worldwide, with the representation of women nearly doubling in many countries [1]. This demographic shift influences how health systems allocate resources and organize care, leading to increased attention on patient outcomes by physician sex. Evidence from high-income countries suggests that female physicians may achieve equal or better outcomes than their male counterparts across measures such as, mortality, readmission, adherence to clinical guidelines, and patient satisfaction [2–16]. While these differences may be explained by mechanisms such as communication style, patient trust, and variations in clinical decision-making, most evidence is drawn from Western health systems. Low- and middle-income country contexts remain largely overlooked, leaving important gaps in understanding whether observed patterns generalize to settings where healthcare systems, workforce sex composition, and resource constraints differ fundamentally. These unique factors may differently shape how physician sex influences care delivery [17–20].

Surgical care quality and postoperative recovery efficiency are critical to patient well-being and health service delivery [21–23]. Length of stay (LOS) after surgery is a key measure of efficiency and quality of care [24, 25]: it reflects the speed of patient recovery, which then influences hospital bed availability, and signals broader resource use [26–30]. While prior research on physician sex has focused primarily on mortality, 30-day readmission, and complications, LOS remains understudied as a primary endpoint, which represents factors unrelated to complications or illness severity [28]. This gap is especially important in LMICs, where prolonged hospitalization can impose substantial financial and opportunity costs on families, contribute to hospital overcrowding, and delay access for others [17].

Our study makes several contributions to the literature. First, we provide new evidence from an LMIC by leveraging a large administrative dataset from China to examine the association between surgeon sex and patient outcomes. Only one prior study has examined this topic in an LMIC contexts [31]. 

Second, while a large literature has considered patient–physician sex concordance [14, 32–43], there has been limited examination of the role of team-level sex composition, such as the combination of sexes between a lead clinician and their assistant, on patient outcomes, particularly in LMIC context. In surgical care, where intraoperative teamwork is essential, such sex pairings may affect patient care through their influence on communication, decision-making, procedural efficiency. Existing studies examining sex discordance in physician teams have focused on high-income countries and report mixed results [44–48]. Our study examines the sex pairing of the operating surgeon and first assistant to assess its association with patient recovery, thereby shedding light on how team-level sex composition may shape outcomes in an LMIC setting.

Third, we strengthen the methodological approach used in this literature. Most prior studies adjust for patient and hospital characteristics to account for confounding factors. We go further by incorporating surgery-type and hospital-month fixed effects, enabling comparisons for identical procedures in the same institution and month. This approach reduces bias from unobserved differences in case complexity, patient mix, and institutional factors.

Finally, we explore heterogeneity by surgery type and surgeon birth cohort to identify whether sex-linked differences are concentrated in specific procedures or generational groups. In LMIC settings, there may be cohort effects arising from unequal training opportunities, cultural differences, and shifting sex norms, but they remain an important and largely understudied dimension of the physician sex literature.

By situating our analysis in an LMIC surgical setting, focusing on LOS as a primary outcome, incorporating team-level sex dynamics, and applying a rigorous fixed-effects study design, this study complements and extends the predominantly high-income country, mortality-focused literature on physician sex and patient outcomes.

## Methods

### Data source

We used administrative health data on surgical inpatient records from a large provincial healthcare database in China, covering all surgeries performed between January 1 and December 31, 2023. We collected Electronic Medical Records (EMRs) from general hospitals across all 10 prefecture-level cities in a western province of China. According to confidentiality agreements, the province name cannot be disclosed (hereinafter referred to as Province S). EMRs include patients’ demographics (e.g., age, sex), disease information (e.g., primary diagnosis, ICD-10 code), treatment characteristics (e.g., payment method, admission route, surgical procedure, hospital level, length of stay), and treatment and examination costs (e.g., total and out-of-pocket expenditures). All records were de-identified prior to analysis.

The sex and age of the physicians and surgeons in EMRs were linked from the Health Workforce Basic Information Database (HWBID), which is part of China’s national statistical survey system for health resources and medical services. Records were matched by the name of physician or surgeon and the hospital name. These records also identify the specific personnel assigned to the roles of surgeon and first assistant, which allows us to define those respective roles. The initial dataset after the linkage included 1,600,535 surgical records with complete information on surgeon sex and age.

### Sample selection

To ensure data quality and make the data comparable, several exclusion criteria were applied. First, we excluded rare procedures, defined as surgical categories with fewer than 10,000 cases in 2023, to focus on commonly performed operations. Second, we excluded low-volume hospitals, where records from hospitals with less than 1,000 surgeries during the study period were removed, to ensure hospitals in the sample had substantial surgical activity. Finally, to reduce the impact of extreme values, records with LOS above the 99th percentile (top 1%) were excluded. After applying these criteria, a total of 602,608 surgical records performed by 11,994 surgeons in 107 hospitals in Province S in 2023 were included in the final analysis. The flowchart of sample selection is presented in Additional file 1: Table S1.

### Outcome variable

Our primary outcome was length of stay after surgery (LOSAS), measured in days. For each patient, LOSAS was defined as the number of days from the date of surgery to the date of hospital discharge. LOSAS captures the postoperative recovery period and is widely used as an indicator of surgical outcomes and efficiency of care.

### Independent variables

The main independent variable of interest was the sex of the operating surgeon, coded as a binary indicator (female = 1, male = 0). Each record was linked to the lead surgeon’s identifier, allowing us to determine surgeon sex for every procedure.

We controlled for multiple covariates at the patient, surgeon, and procedure levels. Patient-level covariates include age (in years) and health insurance type (categories such as employee insurance, resident insurance, poverty alleviation subsidy, commercial insurance, public assistance, self-pay, etc.). Surgeon-level covariates included the surgeon’s age, and highest education level (doctoral degree, master’s, bachelor’s, or college diploma or lower).

To evaluate potential cohort effects, we categorized surgeons into five distinct ten-year birth cohorts: 1956–1965, 1966–1975, 1976–1985, 1986–1995, and 1996–2005. These groupings allow for the assessment of generational differences within our heterogeneity analysis.

### Estimation strategy

We estimated the association between surgeon sex and LOSAS using a Poisson distribution model. This model is suited for count data and skewed distributions common in hospital length of stay metrics. We used this approach over a linear model because LOSAS data typically exhibit a significant rightward skew that a Poisson framework can more accurately model. The model specification is:

$$\begin{array}{rcl}\:{Losas}_{ijkt}&=&{\beta\:\bullet\:Surgeon{\prime\:}s\:sex}_{j}+{Z}_{ij}\\&&+{\theta\:}_{k}+{\delta\:}_{ij}+{\mu\:}_{kt}+\epsilon\:\end{array}$$

where, *i* denotes patients, *j* denotes surgeon, *k* denotes hospitals, and *t* denotes month. $\:Surgeon{\prime\:}s\:sex$, an indicator for female surgeon, is the main variable of interest. $\:{Z}_{ij}$ represents the patient-level covariates described earlier. $\:{\theta\:}_{k}$ is the fixed effect of hospital. Surgery-type fixed effects ($\:{\delta\:}_{ij})\:$refer to the individual ICD-10 procedure codes that adjust for case mix, and hospital-month fixed effects $\:\left({\mu\:}_{kt}\right)$ control for institutional differences and temporal variation within hospitals. Standard errors are clustered at the hospital and month level.

This design enables comparisons of patient outcomes across male and female surgeons performing the same types of procedures, in the same hospital, during the same month. By combining patient-, surgeon-, and hospital-level controls with multiple fixed effects, our approach reduces bias from unobserved confounding and isolates the association between surgeon sex and postoperative length of stay.

To address concerns that surgical complexity may bias our estimates, we adjust for several factors. First, we account for the admission route by distinguishing between elective (outpatient), external referral, and emergency department admissions; this helps address the increased urgency and complexity often associated with non-elective cases. Second, we adjust for surgical complexity by including (i) surgery-type fixed effects based on granular ICD-10 procedure codes, (ii) four broad categories of procedures: diagnostic operations, interventional procedures, standard surgical operations, and therapeutic operations, (iii) the total number of surgeries performed during a single admission, and (iv) an indicator for whether multiple procedures occurred on the same day. Together, these covariates account for differences in case intensity and complexity across surgical episodes.

In addition to the primary analysis of the overall sample, we conducted sensitivity analyses across three distinct subsamples: (i) an analysis excluding maternal and child health hospitals, (ii) an analysis restricted to the ten most common surgeries, and (iii) an analysis of balanced surgeries with comparable representation of male and female surgeons. Moreover, we examined potential heterogeneity through secondary interaction analyses between surgeon sex and patient sex, first assistant sex, and surgeon birth cohort. Finally, we have used a negative binomial model as a sensitivity analyses, and those results are presented in the supplementary materials.

## Results

### Sample characteristics

As shown in Additional file 1: Table S2, among patients, 60.3% were female and the mean age in the overall sample was 52.5 years (SD 17.9). Male patients were older on average than female patients (58.8 vs. 48.4 years). Most patients were insured through the resident or employee insurance programs (41.4% and 29.7%, respectively), while 10.9% were self-pay and 16.8% were covered by other programs.

Most admissions (73.3%) originated from outpatient departments, while 21.6% came through emergency departments. Surgical procedures were categorized as standard surgery (39.8%), therapeutic operations (38.9%), diagnostic operations (18.0%), and interventions (3.3%). Patients underwent an average of 1.9 procedures per admission (SD 1.3), and 88.0% of procedures were completed on the same day. The majority of operations (80.1%) took place in tertiary hospitals.

Among surgeons, 58.7% were female. The mean age was 37.9 years (SD 7.8) and mean years of practice was 13.0 years (SD 8.6). Most surgeons held a master’s (54.7%) or doctoral degree (27.7%). The largest birth cohort was those born between 1986 and 1995 (48.5% of surgeons). Additional surgeon characteristics are provided in Additional file 1: Table S3.

### Distribution of surgeries and LOSAS

Additional file 1: Figs. S1–S4 illustrate the distribution of surgeries and postoperative LOSAS by surgeon sex. Female and male surgeons performed surgeries across a wide range of surgery types.

The left panel of Additional file 1: Fig. S1 shows the number of surgery types performed per admission by surgeon sex. For one to four surgery types, more female surgeons conducted the procedures, reflecting their greater representation overall. For admissions with five to seven surgery types, male surgeons were more frequently represented. The right panel of Additional file 1 Fig. S1 shows the distribution of specific surgical procedures by surgeon sex. While the overall distribution was broadly similar across most surgery types, several procedures exhibited clear gender concentration. Female surgeons were substantially more likely to perform childbirth-related operations, such as low cervical cesarean section (surgery code 74.1x) and repair of other obstetric lacerations (code 75.69). In contrast, male surgeons were more commonly responsible for coronary angiography (code 88.55), cerebral angiography (code 88.41), laparoscopic cholecystectomy (code 51.23), and laparoscopic appendectomy (code 47.01). These patterns suggest some degree of gendered distribution of surgical practice areas within the sample.

Additional file 1: Fig. S2 compares the distributions of total LOS and LOSAS by surgeon sex. In both groups, most patients had short or moderate hospital stays (10 days or less), while a smaller fraction stayed much longer, creating a long right-hand tail. Female surgeons had relatively more patients with shorter LOSAS of 1–5 days compared to male surgeons, whereas male surgeons had more patients with longer LOSAS of 6–9 days. Similarly, female surgeons had a greater fraction of patients with shorter LOS of 1–7 days, while male surgeons had a greater fraction of patients with longer LOS of 8–17 days.

Additional file 1: Fig. S3 plots LOSAS jointly against the number of surgeries within the same admission (panel A) and by surgery code (panel B), stratified by surgeon sex. LOSAS increased with the number of surgeries per admission for both sex groups. For one to four surgeries, male surgeons had slightly longer LOSAS; for five to seven surgeries, female surgeons had longer LOSAS. When stratified by surgery code, LOSAS varied as expected, but with no single surgery code accounting for systematic large differences in LOSAS.

Additional file 1: Fig. S4 shows LOSAS distributions across the number of surgeries per admission for the four procedure categories. Within each category, LOSAS distributions overlapped substantially between female and male surgeons, with similar medians. For most procedure types (standard surgery, diagnostic, and therapeutic) LOSAS increased with the number of surgeries per admission. The largest increases were observed for therapeutic operations, where LOSAS rose from 3.4 to 3.2 days for male and female surgeons, respectively, with a single surgery, to 11.2 and 9.6 days for male and female surgeons, respectively, with seven surgeries.

Overall, Additional file 1: Figs. S1–S4 indicate broad similarity in unadjusted distributions of case volume and LOSAS by surgeon sex, with expected variation across procedure counts and procedure categories.

### Main results

**Fig. 1:**
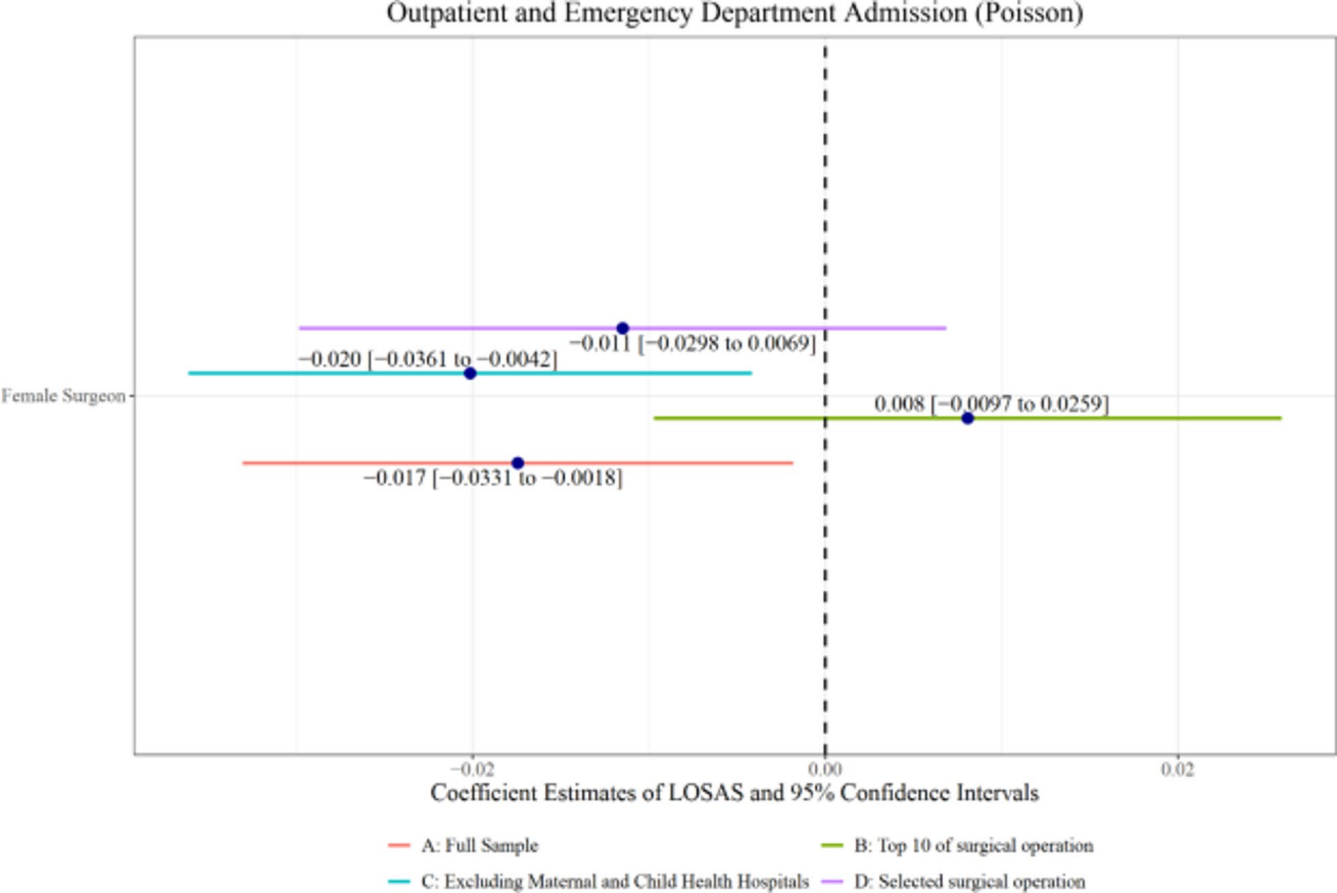
Poisson coefficients for length of stay after surgery using full analysis sample and three subsamples. Note: **Legend A**: Full sample, **Legend B**: Subsample of Top 10 Surgeries including the ICD-10 surgical codes 13.41 (Phacoemulsification and aspiration of cataract), 14.79 (Other vitreous surgery), 33.24 (Bronchoscopy with biopsy), 45.43 (Other endoscopic destruction or excision of large intestine lesion), 51.23 (Laparoscopic cholecystectomy), 74.1x (Low cervical cesarean section), 75.69 (Repair of other obstetric lacerations), 88.55 (Single-catheter technique for coronary angiography), 99.25 (Injection or infusion of cancer chemotherapeutic substance), 99.28 (Injection or infusion of anti-tumor drugs as a biological response modifier), **Legend C**: Subsample after Excluding records from Maternal and Child Health Hospitals, **Legend D**: Subsample of Selected surgical operation, the ICD-10 surgical codes of selected surgical operation are 99.92 (Injection or infusion of other therapeutic or prophylactic substance), 99.28 (Injection or infusion of anti-tumor drugs as a biological response modifier [BRM]), 99.25 (Injection or infusion of cancer chemotherapeutic substance), 93.90 (Non-invasive mechanical ventilation), 93.35 (Thermotherapy), 45.43 (Other endoscopic destruction or excision of large intestine lesion), 45.42 (Endoscopic polypectomy of sigmoid colon), 45.13 (Other endoscopy of small intestine), 44.13 (Other gastroscopy), 43.41 (Endoscopic excision or destruction of gastric lesion or tissue), 39.95 (Hemodialysis), 36.07 (Drug-eluting coronary-artery stent placement), 33.24 (Bronchoscopy with biopsy), 14.79 (Other vitreous surgery), 13.41 (Phacoemulsification and aspiration of cataract). All the models controlled the covariates (No. of Surgeries at Once Admission, Surgeon’s Age and Education, Surgeries Performed at Same Day, Payment Method, Admission Way, Patients’ Age) and fixed effect (Surgical Code, Hospital, Month, and Surgical Category). The full regression results can be found in Table S4. We also provide the negative binomial regression results in the Additional file 1: Table S5.

Figure 1 presents the primary regression estimates derived from the Poisson model examining the association between surgeon sex and LOSAS. The model controls for patient, surgeon, and hospital-level factors including hospital–month, surgery category, and hospital fixed effects. In the full sample, female surgeons were associated with significantly shorter stays (coefficient = -0.017; SE 0.008). To further quantify this association, we conducted post-hoc analyses to estimate the predicted LOSAS in days based on the Poisson estimates for each gender. These estimates indicate a predicted mean stay of 4.62 days (SE 0.127) for male surgeons compared to 4.54 days (SE 0.141) for female surgeons.

To assess the robustness of our primary findings, we conducted sensitivity analyses across three subsamples. First, because female surgeons disproportionately perform surgeries for women and children, we excluded maternal and child health hospitals. The results remained consistent, yielding negative and significant coefficients (coefficient = -0.020; SE 0.008). When restricting the sample to the ten most common surgeries (coefficient = 0.008; SE 0.009) and balanced surgeries with comparable representation of male and female surgeons (coefficient = -0.011; SE 0.009), the associations were insignificant.

**Fig. 2:**
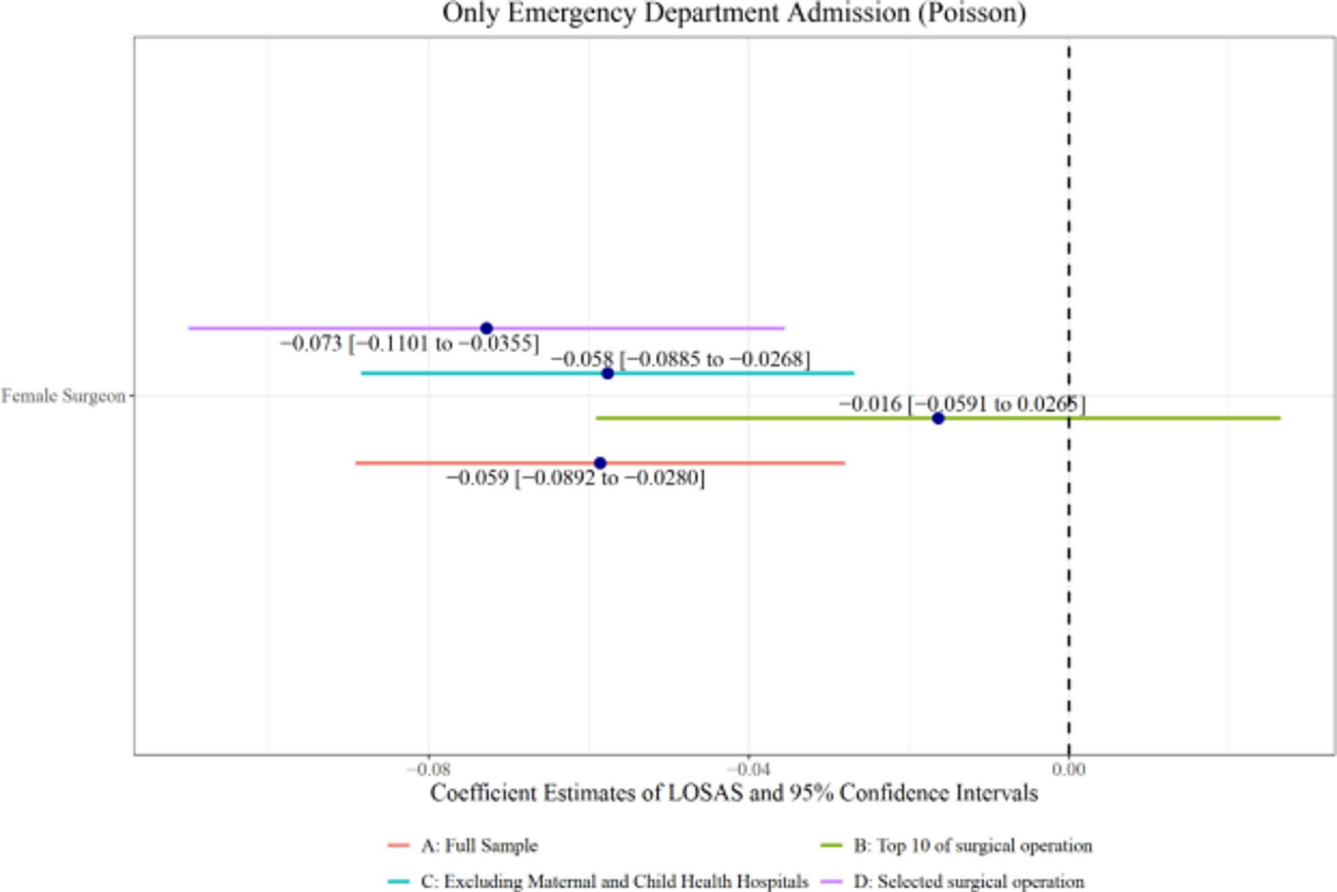
Poisson coefficients for length of stay after surgery using full analysis sample and three subsamples from emergency department. Note: **Legend A**: Emergency department admission (ED admission) sample, **Legend B**: Subsample of Top 10 Surgeries including the ICD-10 surgical codes 13.41 (Phacoemulsification and aspiration of cataract), 14.79 (Other vitreous surgery), 33.24 (Bronchoscopy with biopsy), 45.43 (Other endoscopic destruction or excision of large intestine lesion), 51.23 (Laparoscopic cholecystectomy), 74.1x (Low cervical cesarean section), 75.69 (Repair of other obstetric lacerations), 88.55 (Single-catheter technique for coronary angiography), 99.25 (Injection or infusion of cancer chemotherapeutic substance), 99.28 (Injection or infusion of anti-tumor drugs as a biological response modifier), **Legend C**: Subsample after Excluding records from Maternal and Child Health Hospitals, **Legend D**: Subsample of Selected surgical operation, the ICD-10 surgical codes of selected surgical operation are 99.92 (Injection or infusion of other therapeutic or prophylactic substance), 99.28 (Injection or infusion of anti-tumor drugs as a biological response modifier [BRM]), 99.25 (Injection or infusion of cancer chemotherapeutic substance), 93.90 (Non-invasive mechanical ventilation), 93.35 (Thermotherapy), 45.43 (Other endoscopic destruction or excision of large intestine lesion), 45.42 (Endoscopic polypectomy of sigmoid colon), 45.13 (Other endoscopy of small intestine), 44.13 (Other gastroscopy), 43.41 (Endoscopic excision or destruction of gastric lesion or tissue), 39.95 (Hemodialysis), 36.07 (Drug-eluting coronary-artery stent placement), 33.24 (Bronchoscopy with biopsy), 14.79 (Other vitreous surgery), 13.41 (Phacoemulsification and aspiration of cataract). All the models controlled the covariates (No. of Surgeries at Once Admission, Surgeon’s Age and Education, Surgeries Performed at Same Day, Payment Method, Admission Way, Patients’ Age) and fixed effect (Surgical Code, Hospital, Month, and Surgical Category). We also provide the negative binomial regression results in in Additional file 1: Fig. S5.

Since female surgeons disproportionately perform childbirth-related procedures (Fig. S1), a potential concern is that they may be concentrated in routine cases with fewer complications. To address this, we examined LOSAS exclusively for emergency department admissions (Fig. 2). For the full sample of emergency admissions, the association between female surgeon sex and LOSAS remained negative and statistically significant (coefficient = -0.059; SE 0.015). Post-hoc analyses indicate a predicted mean LOSAS of 4.79 days (SE 0.280) for male surgeons compared to 4.52 days (SE 0.316) for female surgeons. These results remained robust to the exclusion of maternal and child health hospitals (coefficient = -0.058; SE 0.016) and when restricting the analysis to balanced surgeries (coefficient = -0.073; SE 0.019). Lastly, for the ten most common surgeries, the model yielded negative but insignificant results (coefficient = -0.016; SE 0.022).

**Fig. 3:**
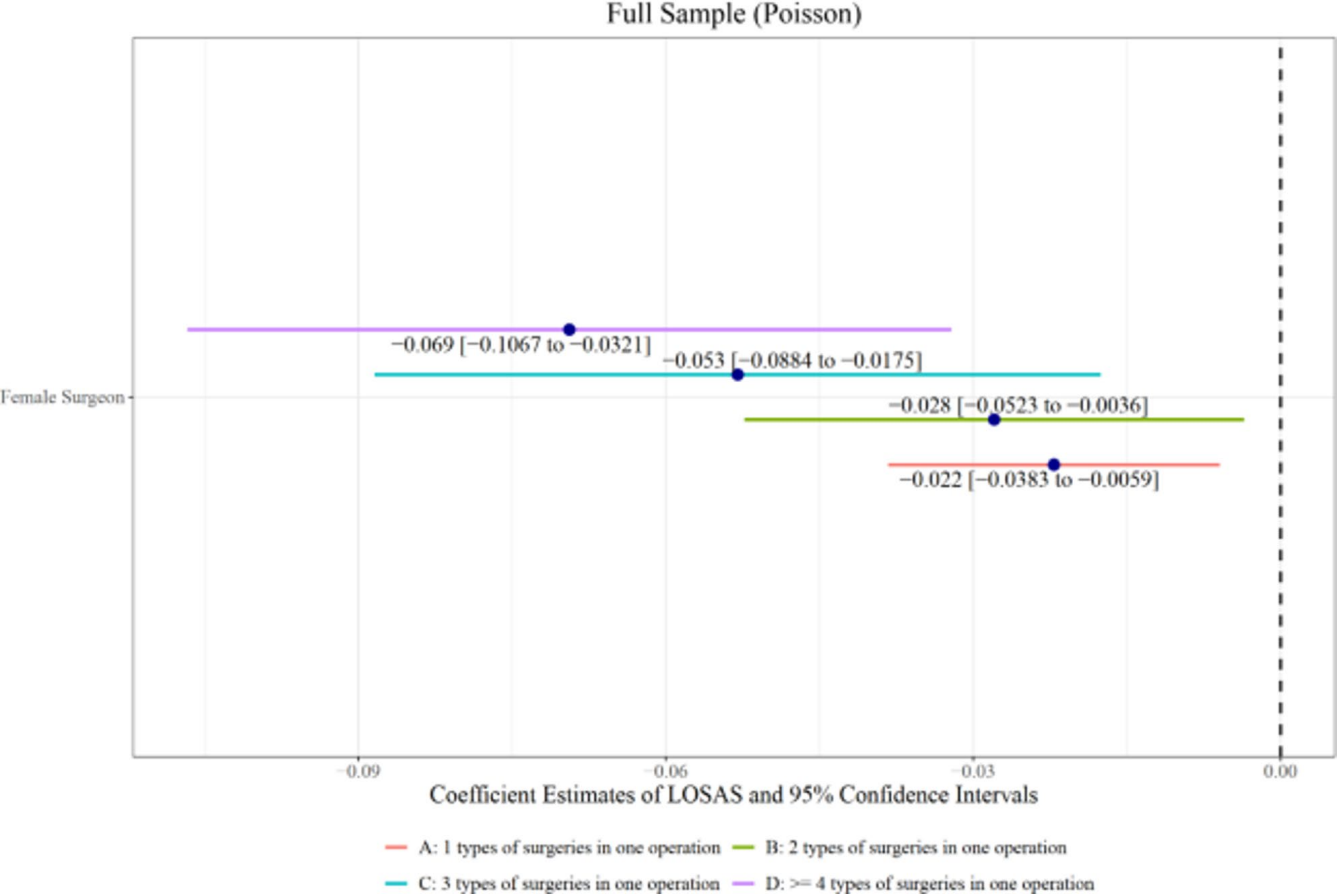
Coefficients for length of stay after surgery using full analysis sample, group by types of surgeries in one operation. Note: The negative binomial regression results can also be found in Additional file 1: Fig. S6.

To further evaluate whether results vary by case complexity, we examined the association based on the total number of surgeries performed during a single admission in Fig. 3. Across all counts—including one, two, three, or four or more surgeries—female surgeons were associated with significantly shorter hospital stays. Post-hoc analyses indicate that the magnitude of the gender difference in LOSAS increased with surgical complexity. For admissions involving one or two procedures, the predicted mean LOSAS were 4.08 (SE 0.144) and 4.26 days (SE 0.197) for male surgeons, compared to 3.99 (SE 0.150) and 4.15 days (SE 0.216) for female surgeons, respectively. However, these differences were more pronounced for higher surgery counts. For admissions involving three procedures, the predicted LOSAS was 4.63 days (SE 0.324) for male surgeons and 4.39 days (SE = 0.329) for female surgeons. For cases involving four or more procedures, the predicted mean stay was 5.17 days (SE 0.394) for male surgeons compared to 4.83 days (SE = 0.419) for female surgeons.

**Fig. 4:**
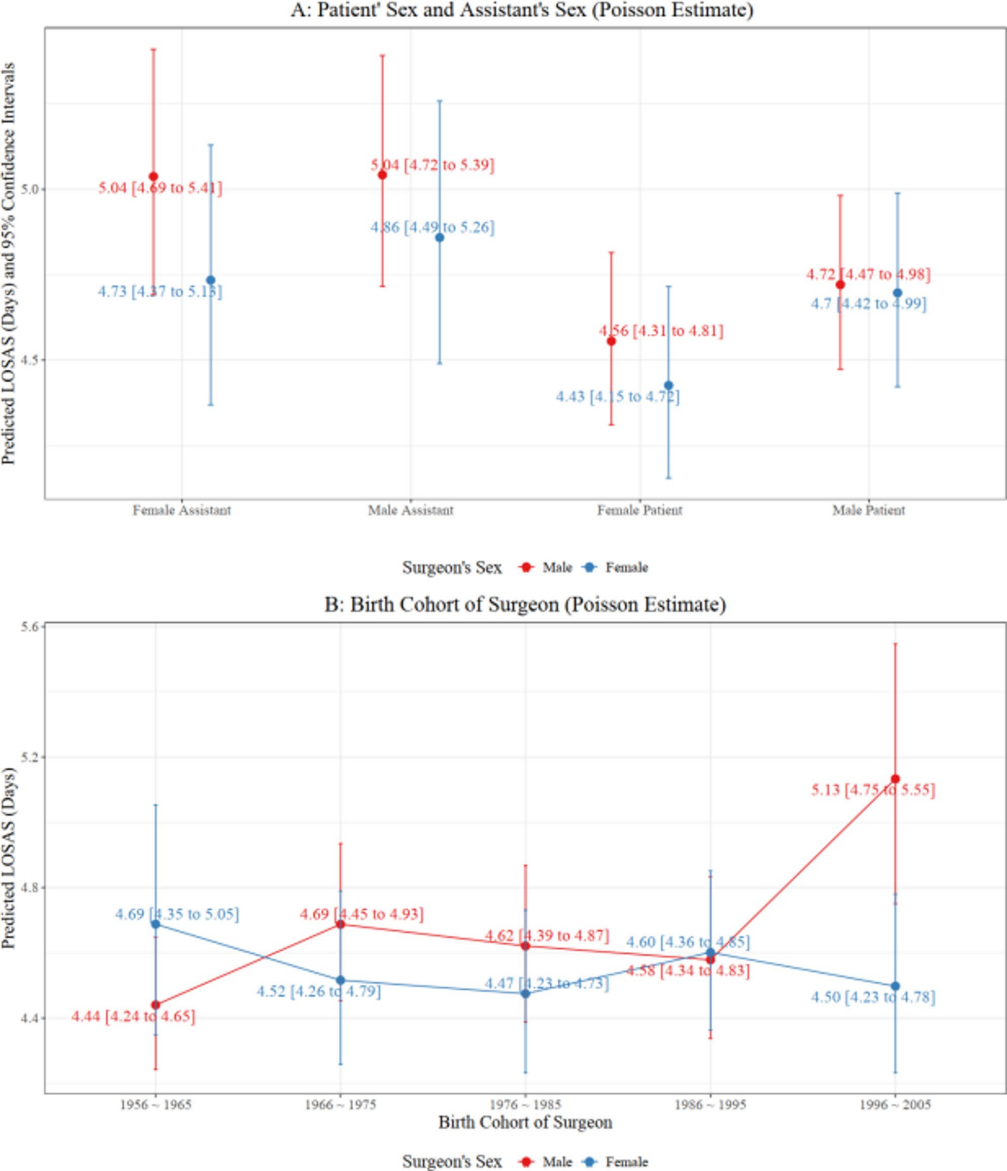
Post-hoc poisson estimate for length of stay after surgery of interaction effect between surgeon’s sex and patient’s sex, assistant’s sex, and birth cohort of surgeon using full analysis sample. Note: The post-hoc estimates for length of stay after surgery were estimated by ‘**fixest (v0.13.2)**’ and ‘**emmeans (v2.0.1)**’ package in R 4.5.2. The detailed results can be found in Additional file 1: Table S6.

Next, we examined heterogeneity through post-hoc estimates of interaction effects between surgeon sex and patient sex, assistant sex, and birth cohort. These results are presented in Fig. 4. For patient-surgeon sex concordance, teams consisting of a female surgeon and a female patient (FF) were associated with the shortest predicted LOSAS at 4.43 days (SE 0.143). In comparison, the predicted stay for male surgeons and male patients (MM) pairing was 4.72 days (SE 0.130). The difference in LOSAS between the FF and MM groups is statistically significant.

For surgeon–assistant sex concordance, teams consisting of a female surgeon and a female first assistant (FFA) were associated with the shortest LOSAS of 4.73 days (SE 0.194). In contrast, both the male surgeon and male assistant pairing (MMA) and the male surgeon and female assistant pairing (MFA) were associated with a significantly longer LOSAS of 5.04 days (SE 0.172), respectively.

Finally, when examining surgeon birth cohorts, the interaction terms (sex × cohort) indicate no significant differences in LOSAS for female surgeons from older cohorts born between 1956 and 1995. However, for the youngest cohort born between 1996 and 2005, female surgeons were associated with significantly shorter LOSAS of 4.50 days (SE 0.204) compared to 5.13 days (SE 0.140) for their male counterparts.

## Discussion

This study examined whether the sex of surgeons is associated with postoperative LOSAS in a large administrative dataset from Chinese hospitals. Leveraging detailed patient-, surgeon-, and hospital-level information, and adjusting for a wide range of covariates and fixed effects, we found that patients with female surgeons had significantly shorter LOSAS relative to those treated by male surgeons. These findings were consistent across the majority of our specifications. The results remained robust when accounting for case complexity, including emergency department admissions and variations in the number of surgeries per admission. Our findings contribute to the growing literature on physician sex and patient outcomes. Prior studies from high income countries generally show that female physicians and surgeons achieve similar or better patient outcomes compared with their male counterparts [2–16]. The only other study from an LMIC context, also in China, found no gender differences in patient readmission rates [31].

At the same time, we observed important patterns: female–female surgeon–patient pairs were also linked to shorter LOSAS relative to male–male pairs; similarly procedures performed by teams with a female surgeon and a female first assistant were associated with shorter LOSAS. The latter result on surgeon-assistant pairing underscores the importance of team dynamics. This adds to emerging evidence that shows that inclusion of women can improve group performance by increasing social perceptiveness and sensitivity within the group, which in turn fosters better collaboration, communication and outcomes [49, 50]. This difference in results by team gender may suggest that traditional gendered hierarchies may influence intraoperative efficiency. In some clinical settings, established social norms could potentially limit communication or psychological safety when a female assistant is paired with a male surgeon. Conversely, female-led teams may experience a more fluid exchange of information and greater mutual trust, which allows for intraoperative efficiency and may help explain the observed improvements in recovery. While our administrative data do not allow for a direct test of these behavioral factors, these findings provide an empirical foundation for future research to investigate how gendered social dimensions influence surgical performance in LMIC contexts.

We also observed generational differences. While there were no significant differences in LOSAS among female surgeons from older cohorts (born 1956–1995), younger female surgeons (1996–2005) were associated with significantly shorter LOSAS. In contrast, younger male surgeons in the same cohort were linked to longer stays, suggesting that cohort-related dynamics may operate differently by gender. These results contrast with findings from the US, where patient mortality generally decreases with both male and female surgeons’ age [15]. This highlights how surgical outcomes in LMICs may be shaped by different contextual factors, and further research is needed to clarify the mechanisms underlying these generational differences.

Taken together, the results suggest that while surgeon sex is associated with shorter LOSAS, the interaction between surgeon sex, team composition, and birth cohort also significantly influences outcomes. For health policy, this highlights the value of moving beyond simple comparisons of male versus female surgeons to a more detailed understanding of workforce diversity and teamwork. In LMIC settings such as China, where hospital bed constraints and financial burdens of prolonged stays are particularly acute, fostering female-led surgical teams may support efficiency in service delivery.

This study possesses several methodological strengths. First, we utilize one of the largest datasets to date—comprising over 600,000 surgical cases—and the largest from an LMIC setting to our knowledge. This substantial sample size enhances statistical power, enabling nuanced subgroup analyses and robust inferences that may not be detectable in smaller studies.

Second, our analytical approach addresses potential confounding by incorporating surgery-type and hospital-month fixed effects, a more granular level of control than is typically found in the literature. We further account for case complexity by including indicators for the total number of surgeries per admission, whether multiple procedures occurred on the same day, and emergency department status. By isolating these variations, our design offers a rigorous test by accounting for both contextual and procedural factors.

One concern may be that female surgeons might be concentrated in specific specialties, such as gynecology, where routine cases with fewer complications could drive the observed results. We address this potential bias by examining LOSAS exclusively for emergency department admissions. These results remained robust across most models, including the full emergency sample, the sample excluding maternal and child health hospitals, and the analysis of balanced surgeries.

Furthermore, we used the total number of surgeries performed during a single admission as another proxy for case complexity. The negative association between female surgeons and LOSAS persisted across all counts, including one, two, three, or four or more surgeries. Collectively, these findings suggest that the shorter hospital stays associated with female surgeons persist regardless of surgical complexity and are not confined to single-procedure cases.

Several limitations warrant caution in interpretation. First, LOSAS, while widely used in administrative and health services research, is an imperfect proxy for surgical quality and patient recovery. Second, this study is descriptive in nature and does not necessarily demonstrate causality. Despite adjustment for patient, surgeon, and hospital characteristics and inclusion of various fixed effects, unobserved confounding remains possible, particularly regarding case severity and intraoperative complexity. Third, while the dataset was large and covered multiple hospitals, the results may not generalize to all of China or to other LMIC contexts. Fourth, some subgroup findings, such as the cohort effects, may be sensitive to sample size and should be interpreted cautiously given sample size constraints.

Future work should build on these findings in several ways. Linking LOSAS to additional outcomes such as postoperative complications, mortality, and readmissions would provide a fuller picture of surgeon sex and team dynamics. Furthermore, future research should move beyond administrative data to incorporate direct observations or qualitative assessments of intraoperative teamwork in the context of LMICs. Such studies could investigate how gendered social dimensions and professional hierarchies influence communication and psychological safety during surgery. Research on intraoperative teamwork, communication, and sexed interactions could help explain the mechanisms underlying observed associations. Comparative studies across different countries and health systems could also shed light on whether the patterns observed here are context-specific or generalizable.

## Conclusions

In conclusion, female surgeons are associated with shorter postoperative length of stay in China. The results were robust in complex scenarios, including emergency department admissions and cases involving multiple surgeries within a single admission. Moreover, team sex composition and generational differences among surgeons significantly influence recovery outcomes. These results suggest that policies to support the integration of female-led surgical teams, such as female surgeon and female first-assistant pairings, can enhance recovery efficiency across various team structures and yield significant benefits for patients and health systems.

## Supplementary Information

Below is the link to the electronic supplementary material.

Supplementary Material 1: Additional File 1: Figures S1–S8, Tables S1–S6.

## Data Availability

The datasets used in this study were obtained from two administrative databases: a patient-level electronic medical record (EMR) database and a surgeon-level health workforce database maintained by the S Provincial Health Commission. These datasets contain individual-level medical and workforce information and are subject to strict confidentiality and data governance requirements. According to the data use agreement between the research team and the S Provincial Health Commission, the data cannot be publicly shared or deposited in open-access repositories due to privacy and legal restrictions. The replication codes used for data processing and statistical analysis can be found at https://shumchi.github.io/notebooks/Composition_and_Patients_LOSAS.html.

## Abbreviations

EMRs: Electronic Medical Records
FF: Female surgeon and female patient
FFA: Female surgeon and a female first assistant
HWBID: Health Workforce Basic Information Database
LMIC: Low- and middle-income country
LOS: Length of stay
LOSAS: Hospital length of stay after surgery
MFA: Male surgeon and female assistant
MMA: Male surgeon and male assistant
