# Supplementary material for: Sex of surgeons and team composition and patients’ length of stay after surgery: evidence from inpatient claims data in China
Source: BMC Med. 2026 Jul 8;24:393. doi: 10.1186/s12916-026-05053-x (PMC13352664; doi:10.1186/s12916-026-05053-x)
Supplement: Supplementary file 1 — Supplementary Material 1: Additional File 1: Figures S1–S8, Tables S1–S6. [file 12916_2026_5053_MOESM1_ESM.docx]

**Supplementary Appendix**

**Appendix Tables**

### Table S1. The flowchart of sample selection

| **Sample selection steps** | **Remained** | **Dropped** |
| --- | --- | --- |
| Raw sample, no missing value on the name, sex and age of surgeon and surgery name | 1,600,535 | 0 |
| Excluded records from surgical categories with <10,000 cases | 660,941 | 939,594 |
| Excluded records from hospitals with <1,000 total surgeries | 608,224 | 52,717 |
| Excluded records above the 99th percentile of LOS | 602,608 | 5,616 |

### Table S2. Demographic description and hospitalization characteristics of patients

| **Characteristics** | **Overall** N = 602,608 | **Male** N = 238,949 | **Female** N = 363,659 |
| --- | --- | --- | --- |
| Age (yr), mean (SD) | 52.5(17.9) | 58.8(16.0) | 48.4(17.9) |
| Payment Type, n (%) |  |  |  |
| Employee Insurance | 179,233.0 (29.7%) | 82,088.0 (34.4%) | 97,145.0 (26.7%) |
| Resident Insurance | 249,285.0 (41.4%) | 95,896.0 (40.1%) | 153,389.0 (42.2%) |
| Poverty Alleviation | 3,973.0 (0.7%) | 1,952.0 (0.8%) | 2,021.0 (0.6%) |
| Commercial Health Insurance | 93.0 (0.0%) | 37.0 (0.0%) | 56.0 (0.0%) |
| Public Expenses | 282.0 (0.0%) | 136.0 (0.1%) | 146.0 (0.0%) |
| Self - payment | 65,931.0 (10.9%) | 18,952.0 (7.9%) | 46,979.0 (12.9%) |
| Social Insurances | 2,646.0 (0.4%) | 955.0 (0.4%) | 1,691.0 (0.5%) |
| Other | 101,165.0 (16.8%) | 38,933.0 (16.3%) | 62,232.0 (17.1%) |
| Admission Type, n (%) |  |  |  |
| Emergency Department | 129,896.0 (21.6%) | 58,680.0 (24.6%) | 71,216.0 (19.6%) |
| Outpatient | 441,482.0 (73.3%) | 167,389.0 (70.1%) | 274,093.0 (75.4%) |
| External Referrals | 787.0 (0.1%) | 370.0 (0.2%) | 417.0 (0.1%) |
| Other | 30,443.0 (5.1%) | 12,510.0 (5.2%) | 17,933.0 (4.9%) |
| Four Types of Surgical Operations, n (%) |  |  |  |
| Diagnostic Operations | 108,320.0 (18.0%) | 60,612.0 (25.4%) | 47,708.0 (13.1%) |
| Intervention | 19,780.0 (3.3%) | 14,779.0 (6.2%) | 5,001.0 (1.4%) |
| Standard Surgery | 239,860.0 (39.8%) | 49,483.0 (20.7%) | 190,377.0 (52.4%) |
| Therapeutic Operations | 234,648.0 (38.9%) | 114,075.0 (47.7%) | 120,573.0 (33.2%) |
| No. of Surgeries at Once Admission, mean (SD) | 1.9 (1.3) | 2.0 (1.4) | 1.8 (1.2) |
| Surgeries Performed at Same Day, n (%) |  |  |  |
| No | 72,478.0 (12.0%) | 30,467.0 (12.8%) | 42,011.0 (11.6%) |
| Yes | 530,130.0 (88.0%) | 208,482.0 (87.2%) | 321,648.0 (88.4%) |
| Quality of Hospital, n (%) |  |  |  |
| Secondary | 119,813.0 (19.9%) | 42,278.0 (17.7%) | 77,535.0 (21.3%) |
| Tertiary | 482,795.0 (80.1%) | 196,671.0 (82.3%) | 286,124.0 (78.7%) |

Note: SD = Standard Deviation

### Table S3. Demographic description of surgeons

| **Characteristics** | **Overall** N = 11,994 | **Male** N = 4,952 | **Female** N = 7,042 | **p-value** |
| --- | --- | --- | --- | --- |
| Age (yr), mean (SD) | 37.9 (7.8) | 40.0 (8.0) | 36.5 (7.3) | <0.001 |
| Year of Working, mean (SD) | 13.0 (8.6) | 14.5 (9.2) | 11.9 (8.0) | <0.001 |
| Education Levels, n (%) |  |  |  | <0.001 |
| Doctoral degree | 3,350.0 (27.7%) | 1,465.0 (29.4%) | 1,885.0 (26.6%) |  |
| Master’s degree | 6,609.0 (54.7%) | 3,008.0 (60.3%) | 3,601.0 (50.8%) |  |
| Bachelor’s degree | 1,878.0 (15.6%) | 465.0 (9.3%) | 1,413.0 (19.9%) |  |
| College diploma or lower | 238.0 (2.0%) | 53.0 (1.1%) | 185.0 (2.6%) |  |
| Birth Cohort, n (%) |  |  |  | <0.001 |
| 1956 ~ 1965 | 235.0 (2.0%) | 149.0 (3.0%) | 86.0 (1.2%) |  |
| 1966 ~ 1975 | 1,365.0 (11.4%) | 781.0 (15.8%) | 584.0 (8.3%) |  |
| 1976 ~ 1985 | 4,041.0 (33.7%) | 1,997.0 (40.3%) | 2,044.0 (29.0%) |  |
| 1986 ~ 1995 | 5,813.0 (48.5%) | 1,920.0 (38.8%) | 3,893.0 (55.3%) |  |
| 1996 ~ 2005 | 540.0 (4.5%) | 105.0 (2.1%) | 435.0 (6.2%) |  |

Note: SD = Standard Deviation; Two Sample t-test and Pearson's Chi-squared test were used to calculate the p-value.

### Table S4. Poisson estimate of coefficients for length of stay after surgery using full analysis sample and three subsamples

| Variables | Full Sample | Subsample | | |
| --- | --- | --- | --- | --- |
|  |  | Top 10 of surgical operation | Excluding Maternal and Child Health Hospitals | Selected surgical operation |
| Surgeon’s sex (Ref: Male) | -0.017** | 0.008 | -0.020** | -0.011 |
|  | (0.008) | (0.009) | (0.008) | (0.009) |
| No. of Surgeries at Once Admission | 0.073*** | 0.076*** | 0.074*** | 0.087*** |
|  | (0.003) | (0.004) | (0.003) | (0.004) |
| Surgeon’s Age | 0.000 | -0.000 | -0.000 | -0.001 |
|  | (0.000) | (0.001) | (0.000) | (0.001) |
| Surgeries Performed at Same Day | -0.252*** | -0.361*** | -0.251*** | -0.290*** |
|  | (0.009) | (0.017) | (0.009) | (0.011) |
| Resident Insurance (Ref: Employee Insurance) | 0.020*** | 0.035*** | 0.020*** | 0.015** |
|  | (0.005) | (0.006) | (0.005) | (0.006) |
| Poverty Alleviation | 0.104*** | 0.088*** | 0.102*** | 0.111*** |
|  | (0.015) | (0.020) | (0.016) | (0.021) |
| Commercial Health Insurance | 0.076 | 0.211 | 0.080 | 0.126 |
|  | (0.104) | (0.169) | (0.107) | (0.193) |
| Public Expenses | 0.001 | -0.103 | 0.006 | -0.022 |
|  | (0.060) | (0.079) | (0.066) | (0.071) |
| Self - payment | -0.023 | -0.042** | -0.031* | -0.081** |
|  | (0.018) | (0.021) | (0.019) | (0.032) |
| Social Insurances | 0.027 | 0.054*** | 0.027 | 0.013 |
|  | (0.017) | (0.018) | (0.017) | (0.025) |
| Other | -0.080*** | -0.122*** | -0.088*** | -0.124*** |
|  | (0.025) | (0.031) | (0.026) | (0.046) |
| Outpatient (Ref = ED) | -0.067*** | -0.048*** | -0.070*** | -0.067*** |
|  | (0.007) | (0.007) | (0.007) | (0.010) |
| External Referrals | 0.063** | 0.190*** | 0.062* | 0.017 |
|  | (0.031) | (0.044) | (0.032) | (0.044) |
| Other | -0.093*** | -0.080* | -0.096*** | -0.067* |
|  | (0.025) | (0.043) | (0.025) | (0.035) |
| Patient’ Age | 0.002*** | 0.000 | 0.002*** | 0.001*** |
|  | (0.000) | (0.000) | (0.000) | (0.000) |
| Num.Obs. | 602608 | 396861 | 585678 | 374728 |
| AIC | 2592324.0 | 1554735.4 | 2528771.8 | 1695412.3 |
| BIC | 2608360.2 | 1569994.2 | 2543741.1 | 1710276.5 |
| RMSE | 2.74 | 2.35 | 2.77 | 3.14 |
| FE: Surgeon’s Education | X | X | X | X |
| FE: Hospital | X | X | X | X |
| FE: Hospital^Month | X | X | X | X |
| FE: Surgical Code | X | X | X | X |
| FE: Surgical Category | X | X | X | X |

Note: * *p* < 0.1, ** *p* < 0.05, *** *p* < 0.01. Std. Errors are included in brackets. Std. Errors are clustered at hospital and month level. ED = Emergency Department.

The ICD-10 surgical codes of selected surgical operation are 99.92, 99.28, 99.25, 93.90, 93.35, 45.43, 45.42, 45.13, 44.13, 43.41, 39.95, 36.07, 33.24, 14.79, 13.41. The ICD-10 surgical codes of top 10 Surgeries are 13.41, 14.79, 33.24, 45.43, 51.23, 74.1x, 75.69, 88.55, 99.25, 99.28.

### Table S5. Negative Binomial estimate of coefficients for length of stay after surgery using full analysis sample and three subsamples

| Variables | Full Sample | Subsample | | |
| --- | --- | --- | --- | --- |
|  |  | Top 10 of surgical operation | Excluding Maternal and Child Health Hospitals | Selected surgical operation |
| Surgeon’s sex (Ref: Male) | -0.018** | 0.003 | -0.020** | -0.005 |
|  | (0.009) | (0.010) | (0.009) | (0.010) |
| No. of Surgeries at Once Admission | 0.076*** | 0.081*** | 0.078*** | 0.101*** |
|  | (0.004) | (0.005) | (0.004) | (0.006) |
| Surgeon’s Age | 0.000 | -0.001 | -0.000 | -0.000 |
|  | (0.000) | (0.001) | (0.000) | (0.001) |
| Surgeries Performed at Same Day | -0.305*** | -0.404*** | -0.305*** | -0.386*** |
|  | (0.013) | (0.023) | (0.014) | (0.018) |
| Resident Insurance (Ref: Employee Insurance) | 0.026*** | 0.037*** | 0.027*** | 0.030*** |
|  | (0.005) | (0.006) | (0.005) | (0.006) |
| Poverty Alleviation | 0.109*** | 0.096*** | 0.107*** | 0.108*** |
|  | (0.017) | (0.022) | (0.018) | (0.027) |
| Commercial Health Insurance | 0.105 | 0.227 | 0.112 | 0.184 |
|  | (0.115) | (0.189) | (0.118) | (0.223) |
| Public Expenses | 0.005 | -0.086 | 0.012 | -0.015 |
|  | (0.057) | (0.077) | (0.065) | (0.072) |
| Self - payment | -0.034* | -0.056** | -0.043** | -0.123*** |
|  | (0.020) | (0.024) | (0.020) | (0.038) |
| Social Insurances | 0.036** | 0.056*** | 0.036** | 0.032 |
|  | (0.018) | (0.020) | (0.018) | (0.027) |
| Other | -0.118*** | -0.165*** | -0.130*** | -0.241*** |
|  | (0.032) | (0.037) | (0.035) | (0.072) |
| Outpatient (Ref = ED) | -0.071*** | -0.050*** | -0.074*** | -0.077*** |
|  | (0.007) | (0.008) | (0.007) | (0.012) |
| External Referrals | 0.066** | 0.163*** | 0.065** | 0.019 |
|  | (0.030) | (0.041) | (0.030) | (0.043) |
| Other | -0.107*** | -0.107*** | -0.110*** | -0.075** |
|  | (0.025) | (0.041) | (0.025) | (0.036) |
| Patient’ Age | 0.002*** | 0.000 | 0.002*** | 0.001*** |
|  | (0.000) | (0.000) | (0.000) | (0.000) |
| Num.Obs. | 602608 | 396861 | 585678 | 374728 |
| AIC | 2488106.0 | 1513413.5 | 2419595.9 | 1550471.1 |
| BIC | 2504142.2 | 1528672.3 | 2434565.2 | 1565335.3 |
| RMSE | 2.79 | 2.37 | 2.82 | 3.28 |
| FE: Surgeon’s Education | X | X | X | X |
| FE: Hospital | X | X | X | X |
| FE: Hospital^Month | X | X | X | X |
| FE: Surgical Code | X | X | X | X |
| FE: Surgical Category | X | X | X | X |

Note: * *p* < 0.1, ** *p* < 0.05, *** *p* < 0.01. Std. Errors are included in brackets. Std. Errors are clustered at hospital and month level. ED = Emergency Department.

The ICD-10 surgical codes of selected surgical operation are 99.92, 99.28, 99.25, 93.90, 93.35, 45.43, 45.42, 45.13, 44.13, 43.41, 39.95, 36.07, 33.24, 14.79, 13.41. The ICD-10 surgical codes of top 10 Surgeries are 13.41, 14.79, 33.24, 45.43, 51.23, 74.1x, 75.69, 88.55, 99.25, 99.28.

### Table S6. Post-hoc estimate for length of stay after surgery of interaction effect (Poisson)

| Interaction Variable | Sex of Surgeon | LOSAS | SE | Lower 95% CI | Upper 95% CI |
| --- | --- | --- | --- | --- | --- |
| Male Patient | Male Surgeon | 4.72 | 0.130 | 4.47 | 4.98 |
|  | Female Surgeon | 4.70 | 0.144 | 4.42 | 4.99 |
| Female Patient | Male Surgeon | 4.56 | 0.129 | 4.31 | 4.81 |
|  | Female Surgeon | 4.43 | 0.143 | 4.15 | 4.72 |
| Male Assistant | Male Surgeon | 5.04 | 0.172 | 4.72 | 5.39 |
|  | Female Surgeon | 4.86 | 0.196 | 4.49 | 5.26 |
| Female Assistant | Male Surgeon | 5.04 | 0.183 | 4.69 | 5.41 |
|  | Female Surgeon | 4.73 | 0.194 | 4.37 | 5.13 |
| Birth Cohort: 1956 ~ 1965 | Male Surgeon | 4.44 | 0.103 | 4.24 | 4.65 |
|  | Female Surgeon | 4.69 | 0.180 | 4.35 | 5.05 |
| Birth Cohort: 1966 ~ 1975 | Male Surgeon | 4.69 | 0.123 | 4.45 | 4.93 |
|  | Female Surgeon | 4.52 | 0.136 | 4.26 | 4.79 |
| Birth Cohort: 1976 ~ 1985 | Male Surgeon | 4.62 | 0.122 | 4.39 | 4.87 |
|  | Female Surgeon | 4.47 | 0.127 | 4.23 | 4.73 |
| Birth Cohort: 1986 ~ 1995 | Male Surgeon | 4.58 | 0.126 | 4.34 | 4.83 |
|  | Female Surgeon | 4.60 | 0.124 | 4.36 | 4.85 |
| Birth Cohort: 1996 ~ 2005 | Male Surgeon | 5.13 | 0.203 | 4.75 | 5.55 |
|  | Female Surgeon | 4.50 | 0.140 | 4.23 | 4.78 |

### Appendix Figures

###
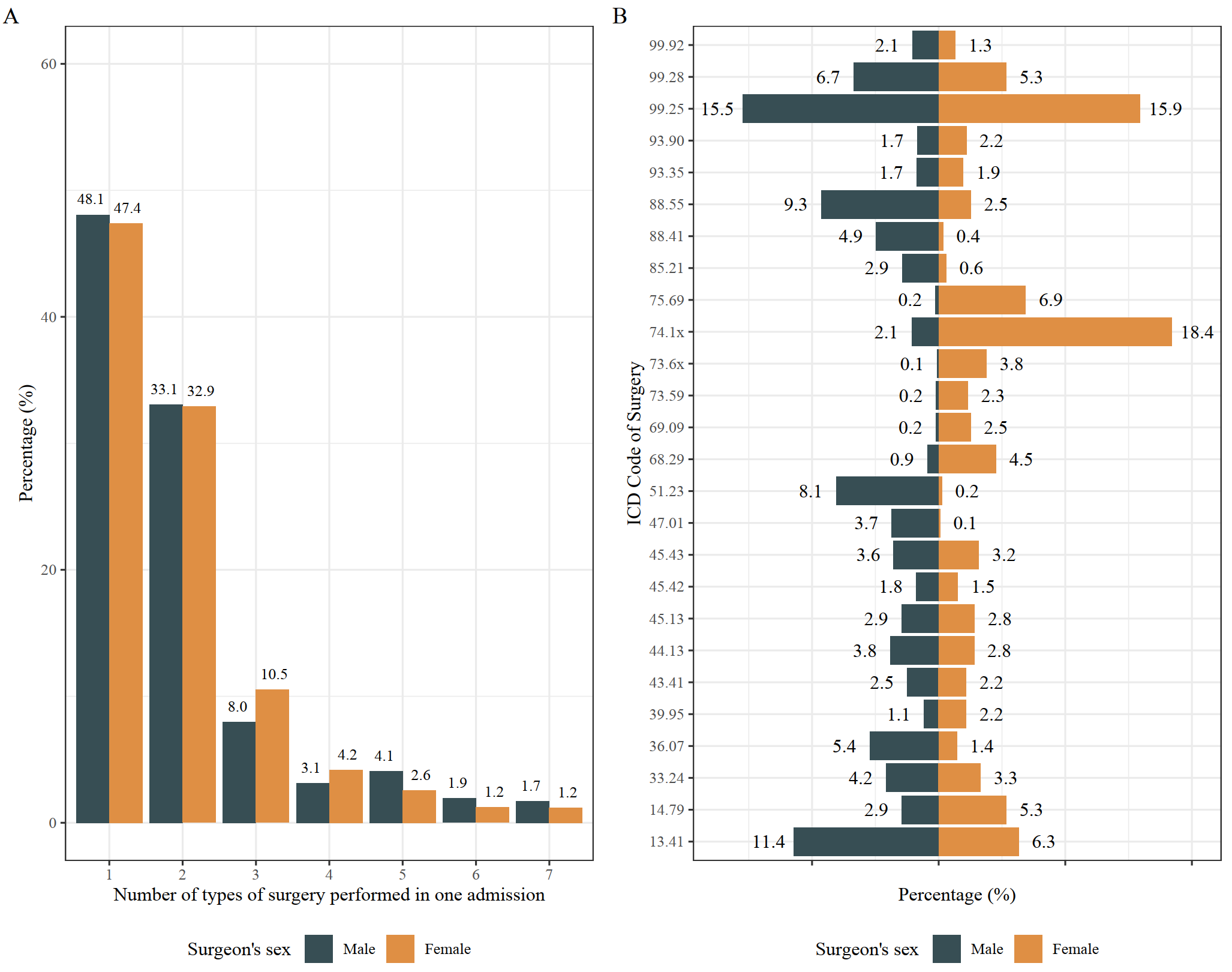


### Fig S1. Distribution of number of surgeries, by surgeon’s sex

Note: The surgical name can be found in the in brackets. 99.92 (**Injection or infusion of other therapeutic or prophylactic substance**), 99.28 (**Injection or infusion of anti-tumor drugs as a biological response modifier [BRM]**), 99.25 (**Injection or infusion of cancer chemotherapeutic substance**), 93.90 (**Non-invasive mechanical ventilation**), 93.35 (**Thermotherapy**), 88.55 (**Single-catheter technique for coronary angiography**), 88.41 (**Cerebral angiography**), 85.21 (**Local excision of breast lesion**), 75.69 (**Repair of other obstetric lacerations**), 74.1x (**Low cervical cesarean section**), 73.6x (**Episiotomy**), 73.59 (**Other manipulation-assisted delivery**), 69.09 (**Other Dilation and Curettage (D&C)**), 68.29 (**Other excision or destruction of uterus lesion**), 51.23 (**Laparoscopic cholecystectomy**), 47.01 (**Laparoscopic appendectomy**), 45.43 (**Other endoscopic destruction or excision of large intestine lesion**), 45.42 (**Endoscopic polypectomy of sigmoid colon**), 45.13 (**Other endoscopy of small intestine**), 44.13 (**Other Gastroscopy**), 43.41 (**Endoscopic Excision or Destruction of Gastric Lesion or Tissue**), 39.95 (**Hemodialysis**), 36.07 (**Drug-eluting coronary-artery stent Placement**), 33.24 (**Bronchoscopy with biopsy**), 14.79 (**Other vitreous surgery**), 13.41 (**Phacoemulsification and Aspiration of Cataract**).

### Fig S2. Distribution of Length of Stay After Surgery (LOSAS) and LOS, by surgeon’s sex

### Fig S3. Distribution of Length of Stay After Surgery (LOSAS) between No. of surgeries and surgery code, by surgeon’s sex

Note: The surgical name can be found in the in brackets. 99.92 (**Injection or infusion of other therapeutic or prophylactic substance**), 99.28 (**Injection or infusion of anti-tumor drugs as a biological response modifier [BRM]**), 99.25 (**Injection or infusion of cancer chemotherapeutic substance**), 93.90 (**Non-invasive mechanical ventilation**), 93.35 (**Thermotherapy**), 88.55 (**Single-catheter technique for coronary angiography**), 88.41 (**Cerebral angiography**), 85.21 (**Local excision of breast lesion**), 75.69 (**Repair of other obstetric lacerations**), 74.1x (**Low cervical cesarean section**), 73.6x (**Episiotomy**), 73.59 (**Other manipulation-assisted delivery**), 69.09 (**Other Dilation and Curettage (D&C)**), 68.29 (**Other excision or destruction of uterus lesion**), 51.23 (**Laparoscopic cholecystectomy**), 47.01 (**Laparoscopic appendectomy**), 45.43 (**Other endoscopic destruction or excision of large intestine lesion**), 45.42 (**Endoscopic polypectomy of sigmoid colon**), 45.13 (**Other endoscopy of small intestine**), 44.13 (**Other Gastroscopy**), 43.41 (**Endoscopic Excision or Destruction of Gastric Lesion or Tissue**), 39.95 (**Hemodialysis**), 36.07 (**Drug-eluting coronary-artery stent Placement**), 33.24 (**Bronchoscopy with biopsy**), 14.79 (**Other vitreous surgery**), 13.41 (**Phacoemulsification and Aspiration of Cataract**).

### Fig S4. Distribution of Length of Stay After Surgery (LOSAS) by times facet with surgery category


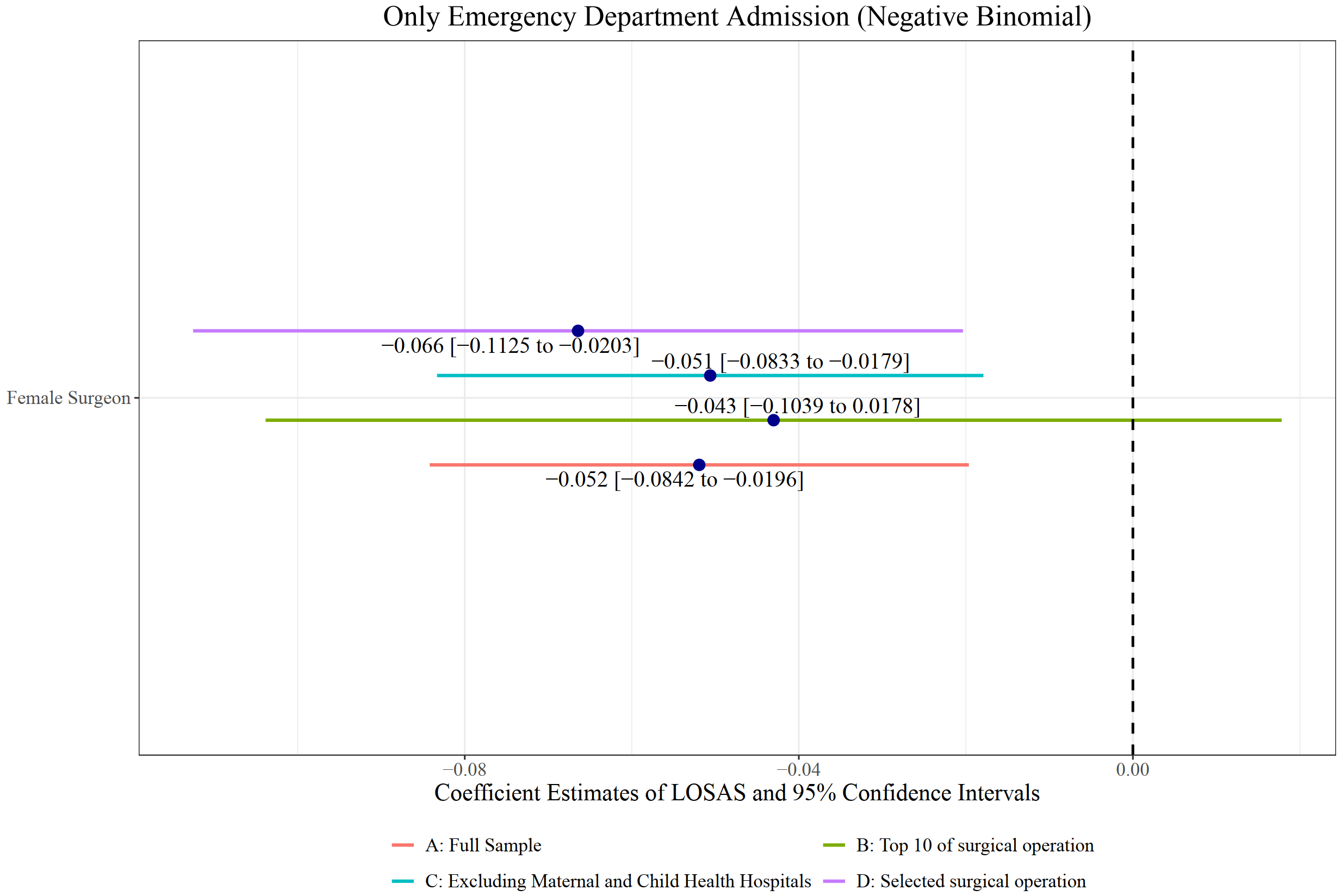


### Fig S5. Coefficients for length of stay after surgery using full analysis sample and three subsamples from emergency department


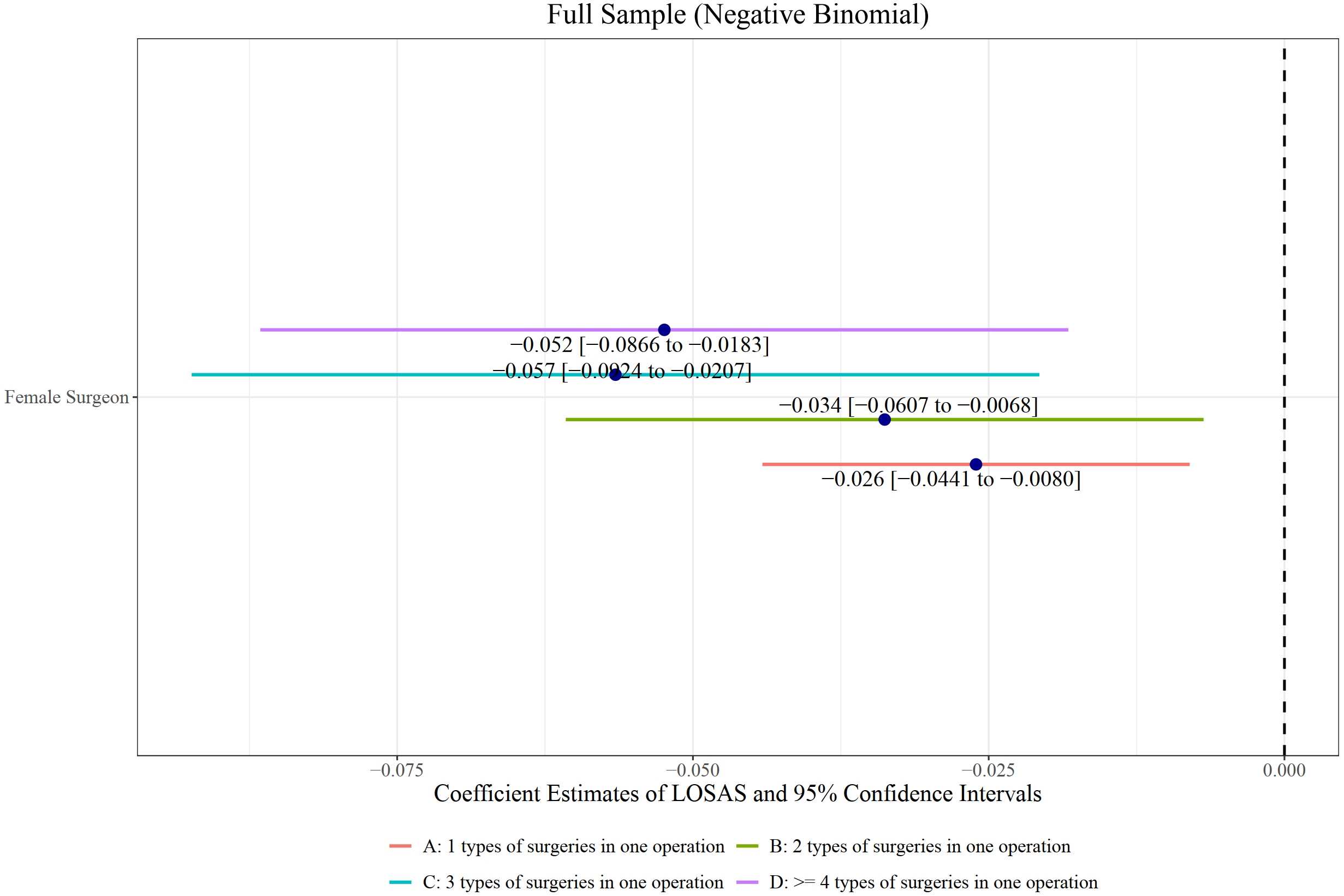


### Fig S6. Coefficients for length of stay after surgery using full analysis sample, group by types of surgeries in one operation


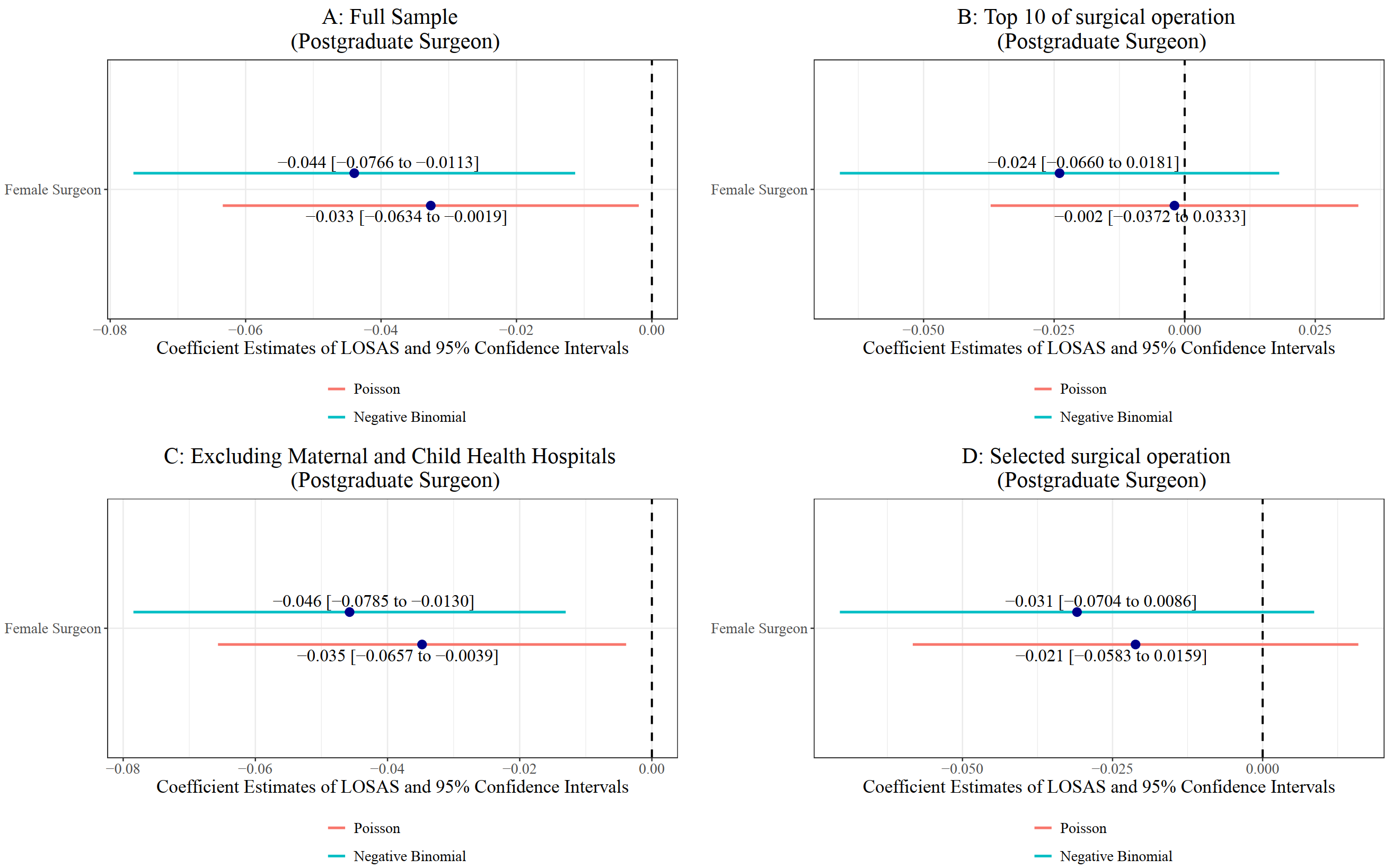


### Fig S7. Coefficients for length of stay after surgery using sample from postgraduate surgeon


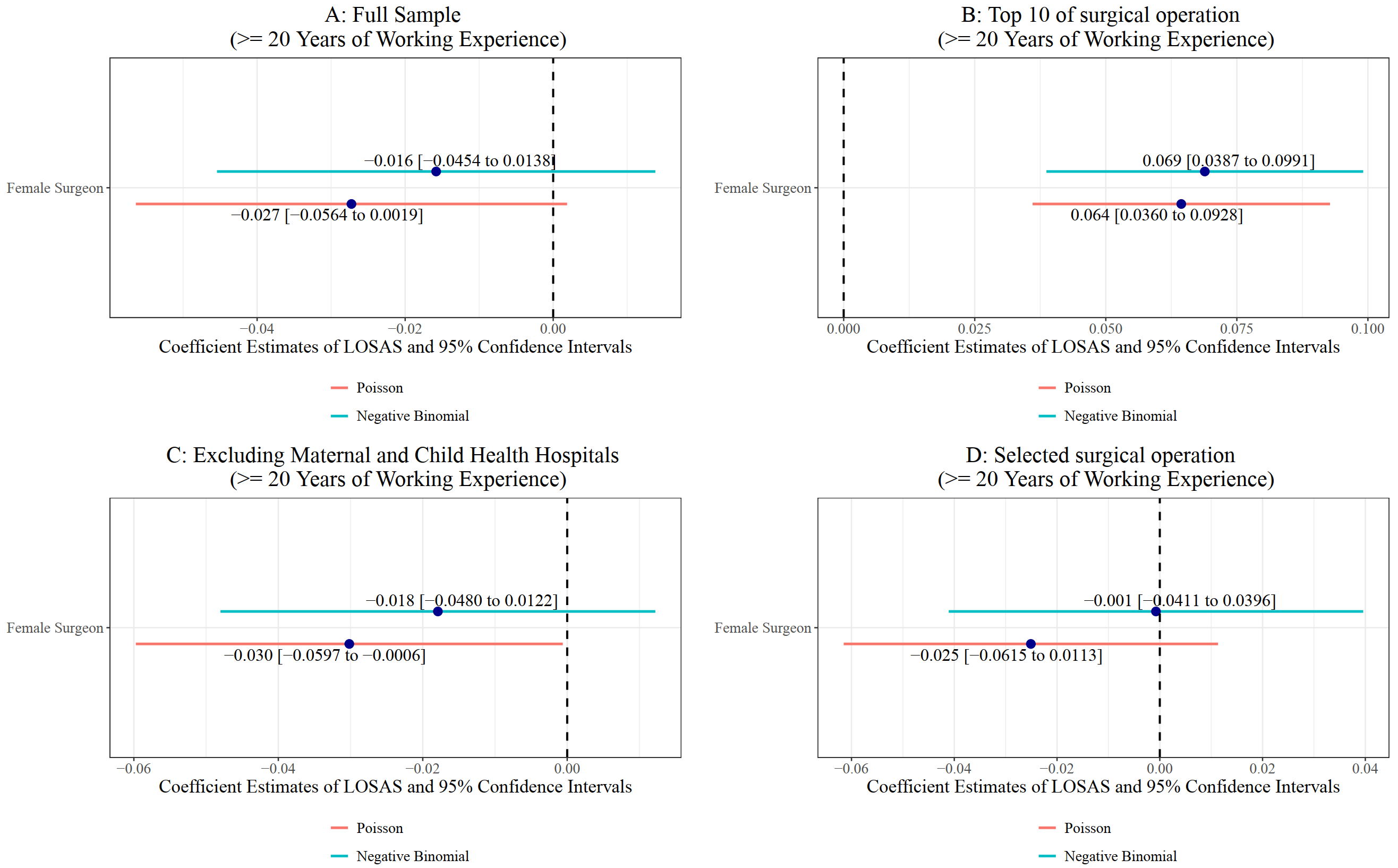


### Fig S8. Coefficients for length of stay after surgery using full analysis sample and three subsamples filtered by year of working experience

Post-hoc of Main regression (Corresponding to the Figure 1)

| Sex of Physician | Day | SE | 95% CI LCL | 95% CI UCL | Sample |
| --- | --- | --- | --- | --- | --- |
| Male | 4.620097 | 0.1269719 | 4.377820 | 4.875782 | A: Full Sample |
| Female | 4.540231 | 0.1408089 | 4.272471 | 4.824772 | A: Full Sample |
| Male | 3.969284 | 0.1778760 | 3.635526 | 4.333684 | B: Top 10 of surgical operation |
| Female | 4.001478 | 0.1966447 | 3.634041 | 4.406067 | B: Top 10 of surgical operation |
| Male | 4.617187 | 0.1299708 | 4.369349 | 4.879083 | C: Excluding Maternal and Child Health Hospitals |
| Female | 4.525138 | 0.1439718 | 4.251576 | 4.816301 | C: Excluding Maternal and Child Health Hospitals |
| Male | 4.844211 | 0.1889401 | 4.487695 | 5.229048 | D: Selected surgical operation |
| Female | 4.788876 | 0.2044709 | 4.404431 | 5.206878 | D: Selected surgical operation |

Post-hoc of ED regression (Corresponding to the Figure 2)

| Sex of Physician | Day | SE | 95% CI LCL | 95% CI UCL | Sample |
| --- | --- | --- | --- | --- | --- |
| Male | 4.793874 | 0.2799344 | 4.275446 | 5.375166 | A: Full Sample |
| Female | 4.521141 | 0.3158312 | 3.942630 | 5.184538 | A: Full Sample |
| Male | 4.065638 | 0.3973901 | 3.356829 | 4.924115 | B: Top 10 of surgical operation |
| Female | 3.999794 | 0.4545519 | 3.201133 | 4.997714 | B: Top 10 of surgical operation |
| Male | 4.770328 | 0.2822092 | 4.248071 | 5.356791 | C: Excluding Maternal and Child Health Hospitals |
| Female | 4.503125 | 0.3186198 | 3.920008 | 5.172982 | C: Excluding Maternal and Child Health Hospitals |
| Male | 5.072907 | 0.3984216 | 4.349149 | 5.917108 | D: Selected surgical operation |
| Female | 4.716822 | 0.4361698 | 3.934939 | 5.654069 | D: Selected surgical operation |

Post-hoc of types of surgeries in one admission (Corresponding to the Figure 3)

| Sex of Physician | Day | SE | 95% CI LCL | 95% CI UCL | Sample |
| --- | --- | --- | --- | --- | --- |
| Male | 4.078970 | 0.1436216 | 3.806970 | 4.370404 | A: 1 types of surgeries in one operation |
| Female | 3.989737 | 0.1499668 | 3.706373 | 4.294764 | A: 1 types of surgeries in one operation |
| Male | 4.263130 | 0.1973491 | 3.893361 | 4.668017 | B: 2 types of surgeries in one operation |
| Female | 4.145615 | 0.2156490 | 3.743783 | 4.590577 | B: 2 types of surgeries in one operation |
| Male | 4.632675 | 0.3235330 | 4.040046 | 5.312236 | C: 3 types of surgeries in one operation |
| Female | 4.393624 | 0.3285925 | 3.794572 | 5.087249 | C: 3 types of surgeries in one operation |
| Male | 5.172010 | 0.3948365 | 4.453257 | 6.006769 | D: ≥ 4 types of surgeries in one operation |
| Female | 4.825187 | 0.4190126 | 4.070024 | 5.720465 | D: ≥ 4 types of surgeries in one operation |
